# Survey of Endodontic Practice amongst Iranian Dentists Participating Restorative Dentistry Congress in Shiraz, November 2007

**Published:** 2008-01-10

**Authors:** Shohreh Ravanshad, Saied Sahraei, Akbar Khayat

**Affiliations:** 1 *Department of Endodontics, School of Dental Medicine, Shiraz University of Medical Sciences, Shiraz, and Iranian Center for Endodontic Research, Tehran, Iran*; 2 *Department of Endodontics, School of Dental Medicine, Shiraz University of Medical Sciences, Shiraz, Iran*

**Keywords:** General Practitioners, Iran, Root Canal Therapy

## Abstract

**INTRODUCTION:** General dental practitioners provide the majority of treatment in Iran. The aim of this study was to gather information on the methods, materials and attitudes employed in root canal treatment by dentists participated in 7^th^ Congress of Iranian Academy of Restorative Dentistry in Shiraz /2007 in order to evaluate the quality of current practice.

**MATERIALS AND METHODS:** A questionnaire for this cross-sectional study was designed with the purpose of evaluating the routine endodontic treatment performed by Iranian dentists. The questionnaire made up of 24 questions with multiple-choice answers. Covering subjects are demographic and professional activity, root-canal preparation and instrumentation, choice of irrigants and disinfectants, and choice of obturation techniques.

**RESULTS:** A total of 247 questionnaires (49.4%) were returned. Ninety-one percent of the respondents were general dental practitioners. The results indicate that there are discrepancies between daily practice and academic teaching, especially regarding the use of rubber dam (only 0.9 % report using it as a standard procedure). Most of practitioners used manual instruments manipulated with a filing technique and few used rotary for canal preparation. The majority of the respondents prepared root canals 0.5-1 mm short of the radiographic apex. The first-choice root-canal irrigant was normal saline (55%), followed by sodium hypochlorite. Approximately, 68% used intracanal medications. The most popular obturation technique was cold lateral condensation (90%) with zinc-oxide eugenol as sealer. Most practitioners performed treatment in two visits for teeth with two or more canals. Eighty-four percent of the dentists used radiograph for determining the working length, and only 2.7% used Apex-locator.

**CONCLUSION:** The survey mentions the importance of continuing dental education for practitioners to update their knowledge.

## INTRODUCTION

Root canal treatment is considered an essential element in the dental services provided to the population in developed countries. Numerous studies have been published evaluating the success and failure of root-canal treatment (1-2). Indeed, many innovative concepts, techniques and instruments have been introduced for the most acceptable cleaning, shaping and obturation. In the past decade, guidelines have been formulated (3) reflecting an increased interest in quality assurance in endodontic procedures. Although the viewpoint of academic teaching and endodontic societies is clear, little information is available regarding the attitude of dental practitioners towards these standards, and on how far the changes in endodontic technique have been incorporated into daily practice. Several studies have revealed that the majority of dentists do not comply with the formulated guidelines on the quality of root canal treatment (2,4-6). These studies investigated the attitude of dentists in Western countries such as Germany (2), Belgium (4), the USA (5), and UK (6). On the other hand, few studies have investigated the attitude of general dental practitioners toward various aspects of endodontic treatment in developing countries (7-9). Epidemiological studies suggest that the failure rate is distinctly higher for teeth treated by dentists who are not endodontic specialists (2,10). However, very few data are available about the general dental practitioners approach to endodontic therapy (5,6,11). These studies mention that a majority of general dental practitioners do not conform to established guidelines. The purpose of this study was to investigate the current opinions of the general dental practitioners in Iran regarding fundamental aspects of routine endodontic treatment and to compare them to academic standards of treatment and established quality assurance guidelines.

**Table 1:** Data related to professional experience of the respondents

**Years of professional experience**	**Frequency**	**Percent**
0-5	114	46.2
6-10	90	36.4
11-20	39	15.8
>20	3	1.2
Missing	1	0.4
Total	247	100

## MATERIALS AND METHODS

A questionnaire was designed for this cross- sectional study with the purpose of evaluating the routine endodontic treatment performed by Iranian dentists. The questionnaire was prepared and piloted by giving it to all endodontic staff of Shiraz University of Medical Sciences. According to replies, the questionnaire was modified. Few questions were added and others were reworded. After evaluating the validity and reliability of the questionnaire, it was randomly distributed to 500 dental practitioners participated in 7^th^ Congress of Iranian Academy of Restorative Dentistry held in Shiraz, November 2007.Respondents were not asked for their names, nor were there any identification numbers, thereby guaranteeing anonymity. The questionnaire was made up of 24 questions with multiple-choice answers. The questionnaire consisted of demographic, professional data and different aspects of endodontic treatment including:

- Root-canal preparation technique and choice of instruments, use of rubber dam, number of appointments, choice of the working length measurement.

- The choice of root-canal irrigant, the concentration of sodium hypochlorite, and the use of intracanal medication.

- The choice of obturation technique and sealer, number of radiographs taken throughout the treatment.

To investigate the influence of the years of practical experience on the materials and techniques employed, the sample was divided into four groups based on the years of professional experience: group 1) up to 5 years; group 2) 6-10 years, group 3) 11-20 years, and group 4) more than 20 years. The collected data were transferred into a personal computer and analyzed using the SPSS statistical package. Simple descriptive statistics were used. The results are given as absolute frequencies and valid percentages in Table 1, Table 2, Table 3, Table 4, Table 5, Table 6.

## RESULTS

From all given questionnaires, 247 filled questionnaires were collected from participants, which show a 49.4 % response rate. The gender of the responding dentist 150 (60.7%) were males and 97 (39.3%) were females. Of these, 225 (91.09%) were general dental practitioners and 22 (8.9%) were specialist. According to working situation 133 (53.8%) were full-time and 114 (46.2%) were part-time. Twenty two practitioners (the specialist) mentioned, they do not perform root-canal treatment. All of the general dental practitioners performed root canal treatment including molar teeth. The distribution of the respondents according to the years of professional experience is shown in Table 1. Distribution of clinical practice duration was not evenly amongst the respondents. The number of the first two groups (0–5 and 6–10) consisted 82.6% of the total respondents due to the significant increase in the number of graduates in the last 10 years.

**Preparation technique: **Majority of respondents (88.4%) indicated that they never isolated the field of operation during root canal therapy with rubber dam; in fact, only four practitioners used rubber dam. The main reason for not using rubber dam was difficulty in using, according to 41% 0f the respondents (Table 4).

**Table 2:** The choice of root canal preparation and instruments

**R** **oo** **t** **canal instrument**	**F** **re** **qu** **e** **n** **cy**	**P** **er** **c** **e** **n** **t**	**Canal preparation technique**	**Frequency**	**P** **e** **r** **c** **e** **n** **t**	**Dist** **a** **n** **c** **e to ** **r** **a** **d** **i** **o** **g** **r** **ap** **h** **ic ** **a** **p** **e** **x**	**F** **re** **qu** **e** **n** **cy**	**Percent**
K-File	68	30.22	Step-back	156	69.3	Flash to apex	24	10.7
K-File, GG drill	96	42.66	Crown down	45	20.0	0.5-1 mm	180	80.0
K-File +Rotary	17	7.55	Step-back + Crown down	24	10.7	>1 mm	5	2.2
NiTi File	25	11.11				According to periapical status	16	7.1
Rotary, GG drill	19	8.44						
Total	225	100	Total	225	100	Total	225	100

**Table 3:** Choice of root-canal irrigants and intracanal-medications

**Root-canal** **irrigants**	**Frequency**	**Percent**	**NaOCl** **concentration**	**Frequency**	**Percent**	**Intracanal medication**	**Frequency**		**Percent**	
Normal Saline	123	54.6	0.5%	41	46.1	Don’t use	71		31.55	
NaOCl	89	39.5	1%	17	19.2	Ca(OH)2	85		37.77	
Chlorhexidine	6	2.7	2.5%	24	26.9	Eugenol	34		15.11	
Distilled water	7	3.1	5.2%	7	7.8	Formocresol	23		10.22	
						CMCP	12		5.33	
Total	225	100	Total	89	100	Total		225		100

**Table 4:** Use of rubber dam and Reasons for not using rubber dam

**Use of Rubber dam**	**Frequency**	**Percent**	**Reasons for not using Rubber Dam**	**Frequency**	**Percent**
Never	199	88.4	Not useful	24	10.66
Occasionally	22	9.8	Patients do not use	34	15.11
Always	4	1.8	Difficult to use	93	41.33
			Additional time	53	23.55
			Extra cost	12	5.33
			Inadequate education	9	4
Total	225	100	Total	225	100

**Table 5:** Working length measurement

**Working length measurement**	**Frequency**	**Percent**
Radiography	187	83.9
Apex locator	6	2.7
Tactile sense	8	3.6
Radio + apex locator	24	10.8
Total	225	100

Majority of the practitioners instrumented the canals using the step-back technique, followed by the crown-down technique, generally using a combination of K file and Gates Glidden burs as instruments. K-files were the most popular instruments. Almost all practitioners prepared canals with hand instruments. Most practitioners (~80%) aimed at achieving a working length between 0.5 and 1 mm short of the radiographic apex (Table 2).

**Canal irrigation solutions and intracanal medicaments: **Over 50% of respondents irrigated root canals with normal saline and 40% used sodium hypochlorite. The most commonly used concentration of sodium hypochlorite was 0.5%, which was used by 46% of the sodium hypochlorite users. The remainder of respondents used either chlorhexidine or distilled water.

Calcium hydroxide was the most common used medicament. The remaining practitioners used different medications including eugenol, formocresol, camphorated monochlorophenol (CMCP) and 32% indicated that they used no intracanal medicaments between appointments (Table 3).

**Figure 1:**
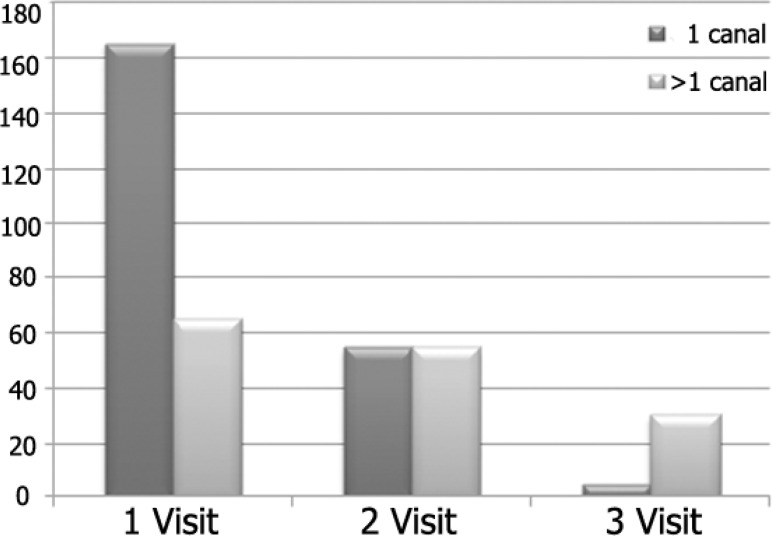
The number of visits according to the number of root canals

**Number of visits to complete root canal treatment: **The number of visits required to complete root canal treatment related to the number of root canals in a tooth is shown in Figure 1. Seventy four percent indicated that they usually completed root canal treatment of single- rooted teeth in one visit. More than half of the respondents indicated that they used two visits to complete treatment of teeth with two or more than one canal, while 30% complete RCT of teeth with two or more canals in one visit and 32 of the respondents complete root canal treatment of these teeth in three visits.

**Table 6:** The choice of obturation technique and type of sealer

**Obturation technique**	**Frequency**	**Percent**	**Type of sealer**	**Frequency**	**Percent**
Lateral	203	90.22	ZOE-based	133	59.11
Vertical	11	4.88	AH26	78	34.66
Single cone	2	0.88	AH plus	5	2.22
Vertical +Lateral	9	4.0	Ca(OH)2-based	9	4
Total	225	100	Total	225	100.0

**Number of radiographs in routine RCT: **Approximately, 40% of practitioners indicated that they took three radiographs (pre-operative, during root canal treatment and post-operative) for routine root canal treatment. Seventy two percent of practitioners took pre-operative radiograph, 80% took radiograph during root canal treatment for determining working length, and only 10% took post-operative radiograph.

**Figure 2:**
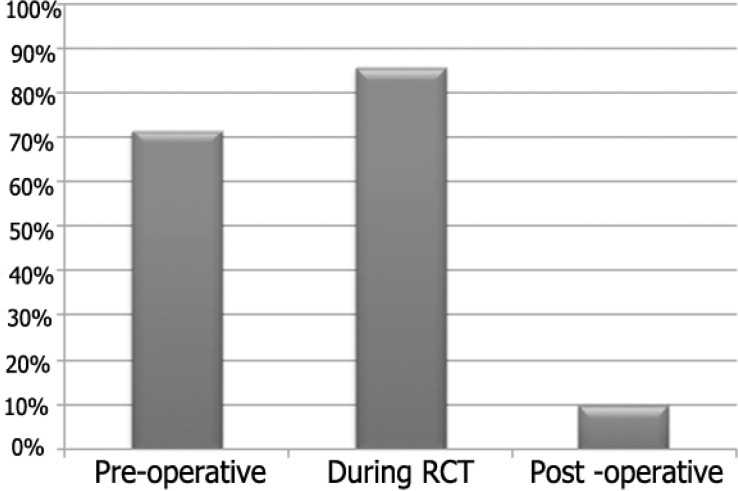
The percentage of practitioners who took radiographs at the various stages of root canal treatment

Figure 2 shows stages of treatment at which radiographs were taken. Most of the practitioners used radiography for working length measurement, only six of the dental practitioner used apex locator and 8 of them used their tactile sense (Table 5).

**Choice of obturation technique: **Cold lateral condensation was the most common obturation technique (90.22%). The majority of dentist reported the use of zinc oxide eugenol based sealer (59%), followed by the AH26 sealer (34.7%). Few dentist (n=9) used calcium hydroxide based sealer. All practitioners used gutta-percha points for obturation (Table 6).

## DISCUSSION

The practitioners selected in this study were the dentists participated in 7th Congress of Iranian Academy of Restorative Dentistry which was held in Shiraz, November 2007, which may not truly be the representatives of the dental population throughout Iran. However, the advantage of using this group was that the information obtained could be related to the teaching of endodontics, the techniques and the materials which were familiar to them. Thus, the information gathered is still important and useful, particularly as it relates to changes that have been introduced in dental practice.

The response rate for this study was low (49.4%) but compares favorably with that of a previous surveys conducted by Jenkins *et al. *obtained a response rate of 41.5% but limited their survey to practitioners graduated from one dental school (6). A similar survey held by the Council of the British Endodontic Society amongst General Dental Practitioners (GDPs) in England also had a low response rate of 32% (12). The response rate of the Flemish dentist survey was 25.1% (4). The majority of respondents were general dental practitioners, reflecting the fact that this is the area where the majority of dental treatment is provided in the Iran.

The step back technique was the most popular canal preparation technique among Iranian general dental practitioners. In another study, 23.6% of Flemish dentists used the step back technique (13). In North Jordan 52.7% GDP used this technique (14). Generally, dentists in Iran tended to use hand instruments and were not inclined to use more advanced engine Driven techniques for shaping the root canal system.

The traditional intracanal instruments such as, K-files together with Gates Glidden drill used in 42.9% by the practitioners. However, 11.2% of the dentists sometimes used nickel–titanium files, indicating that new developments were slowly being incorporated into daily practice. Owing to the variability of the point of exit of the root canal in the apical region (15) determination of the working length has always been a challenge (11). In the study of Sjogren *et al.*, it was stated that in cases where the pulp was necrotic and infected, the working length should be chosen within 1 mm of the radio- graphic apex (1). The optimal working length in teeth with vital pulp appears to be 1-2 mm from the radiographic apex (16). In our survey, 80% of the GDPs prepared canals 0.5-1 mm short of the radiographic apex. Whitten *et al. *(1996) reported that 75% of the respondents stated that they would instrument 0.5 mm short of the radiographic apex (5). In Flemish survey 38.9% of the GDPs used instrumentation levels 1mm short of the radiographic apex, independent of the pathology (4).

Rubber dam isolation is considered the standard of care in endodontics to provide isolation, protection and improve visual access, only 22 dentists reported using rubber dam occasionally and not as a routine practice. Use of rubber dam were found in Sudan (2%) and among Flemish dentists (3.4%) (4,9). A survey amongst American GDPs indicated that 59% always used rubber dam (5) and 57% of general dental practitioners in New Zealand (17). In the UK, 60% to 70% reported not to use rubber dam for any procedure, whereas only 5% of the dentists working principally in the National Health Service (NHS) used rubber dam for endodontic treatment (18-20). The reasons for not using rubber dam could be the extra cost, additional time, lack of adequate skills or training, absence of patient's acceptability or inadequate education in the undergraduate teaching curriculum. In our study the main reason for not using rubber dam was difficult to use (41.33%). The continuing education course attendees should be learned how to use rubber dam.

In the current survey, most dental practitioners used normal saline and sodium hypochlorite solutions as canal irrigants. Sodium hypochlorite is recommended as the material of choice for irrigating the root canal system because of its effective antimicrobial and tissue solving action (21), an opinion that was shared by 39.5% of our respondents. In North Jordan survey, most GDPs used hydrogen peroxide and sodium hypochlorite solutions (14), the same result was demonstrated amongst dentist in Switzerland (22), and in Sudanese's study over 50% of respondents irrigated root canals with hydrogen peroxide and 14% used normal saline (9), while the majority of Flemish respondents (59.2%) used sodium hypochlorite (4). The selection of irrigant could be associated with the use of rubber dam, as it was found that 70% of rubber dam users among British dentists irrigated with sodium hypochlorite, whilst non-users tended to use local anesthetic solution (18). In a study of Whitten *et al*., 79% of the GDPs used sodium hypochlorite as irrigant (5). The current findings do not mirror these findings. The vast majority of our respondents were non-users of rubber dam and more than one third of them (39.5%) use sodium hypochlorite routinely. A similar trend toward using sodium hypochlorite as an irrigant despite not using rubber dam for isolation was noticed amongst Flemish dentists (13). In the UK, the majority of dentists used local anesthetic solution to irrigate the canal space (6). The use of sodium hypochlorite without isolating the field of operation tightly with a rubber dam presents an obviously hazardous practice in the use of potentially irritant irrigation solution. Many clinicians prefer dilute concentrations to reduce the potential of sodium hypochlorite to act as an irritant (23). 46.1% percent of the Iranian GDPs used a concentration of 0.5%, however only 26.9% used concentration of 2.5%, Possibly, the limited use of rubber dam was a factor in the choice of more dilute solutions.

In Slaus and Bottenberg's survey (4) most of the GDPs used the traditional phenol or camphorated products, and only 4% used calcium hydroxide. The same as in Ahmad *et al. *(9), Jenkins *et al. *(6), and Al-omari (14), survey. Despite the fact that calcium hydroxide is recognized as the standard intracanal medicament for inter-appointment dressing, (24) it was used by 37.8% of the present respondents. The use of calcium hydroxide, as intracanal medication, should be encouraged among dentists, as it is effective against most root canal pathogens and able to denature bacterial endotoxins (25-26). 10.2% of the practitioners reported using formocresol. Although it has been used for their antimicrobial and fixative properties, they are toxic to periradicular tissues (27) and may have mutagenic and carcinogenic potential (28).

In the present survey the vast majority of the respondents complete root canal treatment in two visits for teeth with two or more root canals. However, majority of respondents (73.8%) reported completing root canal treatment for teeth with single root canal in one visit. In Sudan, the majority (60%) usually completed root canal treatment in more than three visits (9). In a study demonstrated a clear inclination to single visit endodontics, especially in cases without apical periodontitis (11). Single visit treatment appears to have gained more popularity and an increased credibility in the pre-clinical endodontic teaching in America and Europe (29). Whitten *et al*. found that endodontists preferred single- visit therapy, where as GDPs preferred multiple visits (5). In both cases, the percentage dropped for patients presenting with pain. Most of the GDPs of the Flemish dentists' survey reported little difference in the number of appointments when completing an endodontic treatment in tooth with one or four root canals (4). Multi- visit endodontic treatments could be a direct result of lacking adequate clinical time to complete the treatment in a single visit. The dentists may prefer to wait till the complete subsidence of pain and other symptoms before obturating the canal system. Another possible explanation could be that the initial visit was spent for treating the pain and acute symptoms. As many root canal treatments in general practice occur owing to pulp exposure or acute pain, one session may be spent with an (emergency) pulpotomy with preparation and obturation scheduled for a following appointment. This may explain why there is little difference in the number of sessions between teeth with single and multiple root canals (4).

One objective of root canal treatment is the thorough cleaning and shaping of the root canal system in order to remove bacteria and any organic tissue that may act as a substrate for further bacterial proliferation (30). Correct estimation of the length of the canal(s) is therefore essential and this is usually performed by measuring from a radiographic image of the tooth with an instrument of known length *in situ *(31). In the present study, this was the method of working length estimation favored by the majority of respondents (83.9%). This in agreement with the study performed in UK (6).

Modern electronic apex locators can be accurate, but are often used in conjunction with radiographs because of the additional information about tooth anatomy that a radiograph allows and because it provides a permanent record (32). The use of tactile sensation to determine the working length cannot be recommended, because the instruments may bind against the canal walls at any position along their length, or may perforate apically (33).

The number of radiographs exposed during treatment varied from two to four, with an average of three. In the present survey only 40% took three radiographs, while In Sudanese survey approximately 55% of practitioners indicated that they took three radiographs during root canal treatment, whilst 34% preferred to take two radiographs.

Over the years, numerous methods have been advocated to obturate the prepared root-canal system, each with their own claims of ease, efficiency or superiority. The majority of the general dental practitioners in Iran used cold lateral compaction of gutta-percha to obturate the root canal space. This technique is acknowledged universally and is the most common obturation technique (29). Seemingly, dentists in Iran are not strong advocates of the more recently introduced advanced obturation techniques. This may be attributed to additional cost involved or the lack of skill and training. Pitt Ford *et al *(12) found that in England most private practitioners used non-medicated zinc oxide–eugenol root-canal cements whereas the majority of NHS (National Health Service) practitioners used one particular medicated sealer, Endo-methasone. The most popular root-canal sealer amongst our practitioners was zinc-oxide eugenol (59.6%), although a group of approximately 34.9% used AH26 sealer.

## CONCLUSION

This study investigated the status of endodontic Treatment which is currently practiced by general dental practitioners working in private offices in Iran. It demonstrated that dentists performed procedures with different quality standards, especially in the low use of rubber dam for isolation. General practitioners did not seem to use recently introduced techniques.

Despite a variety of new instruments and techniques, most GDPs used conventional preparation and obturation techniques. Endodontic treatment is still considered to be a tedious procedure for general dental practitioners. Teaching new technology in dental schools and/or continuing education courses is needed nowadays.
